# Horner Syndrome After Anterior Cervical Spine Surgery for Traumatic Spinal Cord Injury – A Rare Complication

**DOI:** 10.7759/cureus.40134

**Published:** 2023-06-08

**Authors:** Shabeeba Sherin P P, Indulekha Shibu, Osama Neyaz, Anshini Gupta

**Affiliations:** 1 Physical Medicine and Rehabilitation, All India Institute of Medical Sciences, Rishikesh, IND

**Keywords:** anterior cervical spine surgery, tetraplegia, trauma, anterior cervical decompression, horner syndrome, spinal cord injury

## Abstract

Horner syndrome (HS) is a rare complication of anterior cervical decompression and fusion procedures (ACDF). A 42-year-old female presented with sudden-onset weakness in both upper and lower limbs secondary to trauma and was diagnosed with a spinal cord injury with tetraplegia. Her pre-operative findings were that her motor level of injury was C4 on the right and C5 on the left, and her sensory level of injury was C4 and C5, respectively, on the right and left sides. Her neurological injury level (NLI) was C4, and her ASIA Impairment Scale score was A. The cervical spine MRI suggested compression fractures of the C5 and C6 vertebral bodies with cord compression. She underwent a C5 and C6 central corpectomy and mesh cage fusion by an anterior longitudinal incision via right-sided exposure. She developed ptosis, miosis, and anhidrosis on the side immediately after surgery. During rehabilitation admission, her neurological findings were that her motor level of injury was C4 on the right and C5 on the left, and her sensory level of injury was C4 and C5, respectively, on the right and left sides. Her NLI was C4, and her ASIA Impairment Scale score was C. Even one year after surgery, the symptoms persisted. HS is a rare complication of anterior cervical spine fixation, and it is essential to have a thorough understanding of intraoperative and postoperative ACDF-related complications to avoid them whenever possible and manage them successfully and safely when they occur.

## Introduction

Anterior cervical decompression and fusion (ACDF) is a frequently used procedure for treating different cervical spine conditions. In this surgical procedure, the cervical spinal cord and nerve root decompression is done in cases of a herniated intervertebral disc, cervical radiculopathy, spondylosis, myelopathy, or deformity by trauma, tumor, or infection [1]. Horner's syndrome (HS) is characterized by ipsilateral pupillary miosis, facial anhydrosis, and ptosis resulting from damage to the cervical sympathetic trunk. HS is a well-known but rare complication of ACDF. In a retrospective, multicenter case series on the complications of anterior cervical spinal surgery, the incidence of HS was estimated to be around 0.06%, while in another case series, the incidence of HS after ACDF was reported to be about 0.45% [2,3]. This rare complication might be caused by damage to cervical sympathetic fibres that run along the medial aspect of the longus colli muscles. The proximity of the cervical sympathetic trunk (CST) to the longus colli muscle, which is often forcefully retracted, sectioned, and cauterized during the surgical approach, makes it vulnerable to injury [4]. This complication leads to functional and cosmetic impairments that can be distressing and financially burden the patient.

## Case presentation

**Figure 1:**
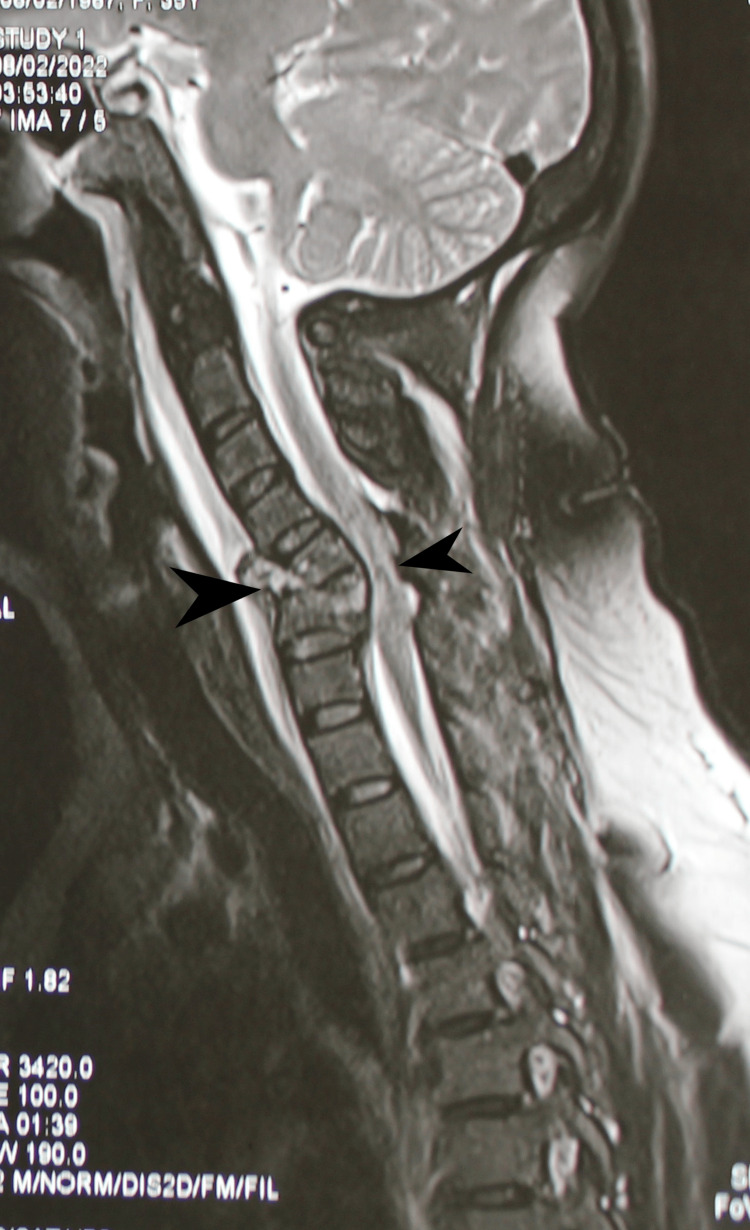
MRI cervical spine showing compression fracture of C5 &C6 vertebra with cord compression

On February 7, 2022, a 42-year-old female homemaker without any previous systemic illness and no family history of neurological disease presented with weakness in all four limbs following a fall from height from the terrace of a building. She suffered a neck injury after sustaining a fall from 12 feet height. She could not sense and move her bilateral upper and lower limbs, because of which she was hospitalized and investigated further on the same day. She was catheterized in the primary hospital because of urinary retention and was referred to a tertiary hospital for further management. In her cervical spine MRI, the T2 weighted sagittal images demonstrate evidence of fracture compression collapse of C5 and C6 vertebral bodies. The posterior part of visualized bodies is retropulsed with the spinal canal causing cord compression. Evidence of ill-defined hyperintensity is seen at this level, suggesting cord oedema. In addition, evidence of a hypointense area within suggests hemorrhagic foci/contusion. The rest of visualized vertebral bodies appear normal, as shown in Figure 1.

**Figure 2:**
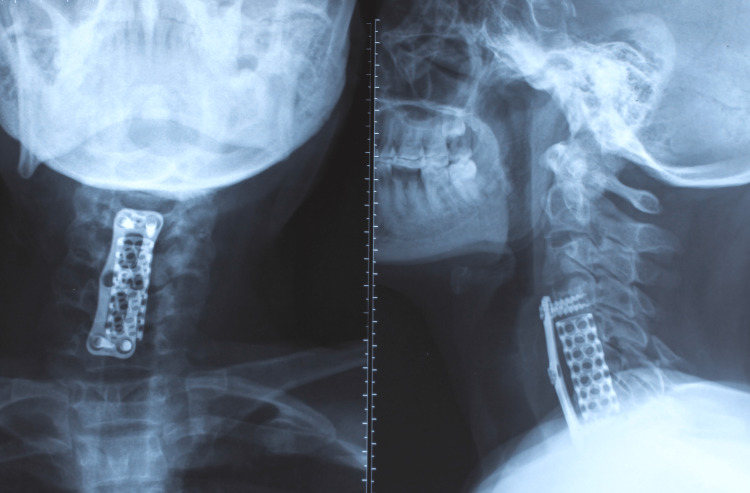
X-ray cervical spine AP & lateral view showing operative fixation

After five days of injury, she was operated on because of an unstable cervical vertebral fracture. Pre-operative, on examination, her motor level of injury was C4 on the right and C5 on the left, and her sensory level of injury was C4 and C5, respectively, on the right and left sides. Her neurological level of injury (NLI) was C4, her American spinal injury association (ASIA) Impairment score was A, and her spinal cord independence measure III (SCIM III) score was 10. On February 12, 2022, with a right anterior approach, a two-inch skin incision was made on the neck at the C5 and C6 vertebrae level to access the injury site. She underwent a C5 and C6 central corpectomy, and anterior cervical stabilization was achieved using a semi-constrained cervical plate and expandable titanium mesh cage (Figure 2). An autogenous ilium bone graft was harvested for grafting. No complications were observed during the operation.

Post-surgically patient developed ptosis of the right upper eyelid and an absence of sweating on the right side of the face. However, this was not a significant concern during the acute postoperative period. However, she later became concerned regarding these symptoms.

**Figure 3:**
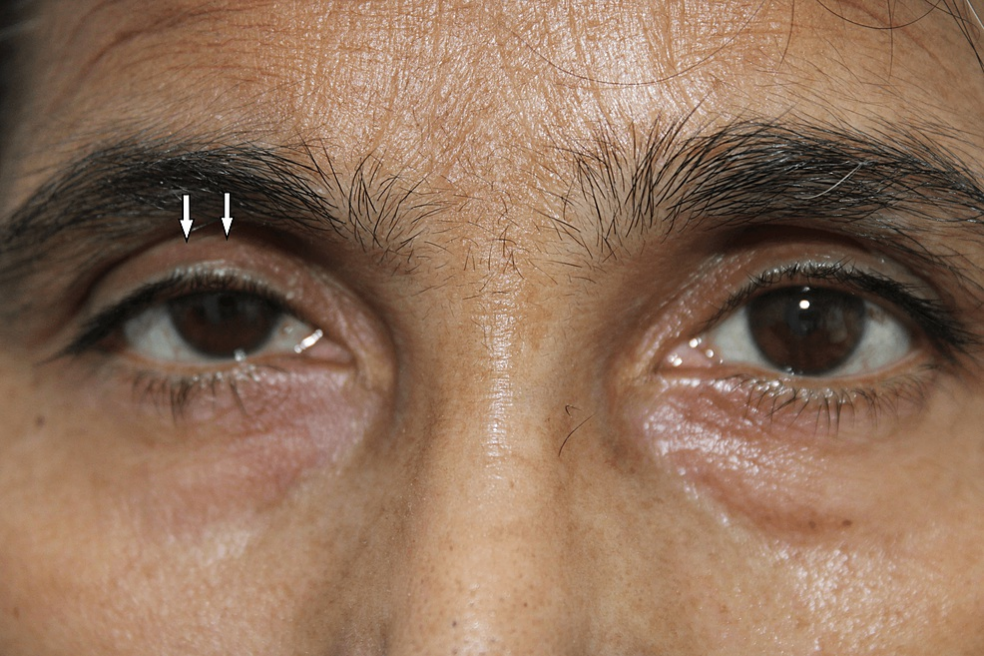
Showing right-sided ptosis

On February 7, 2023, she was admitted for inpatient rehabilitation in the Department of Physical Medicine & Rehabilitation in a tertiary care hospital in Uttarakhand. She was diagnosed with traumatic spinal cord injury with tetraplegia, neurogenic bowel, and bladder. On examination, her motor level of injury was C4 on the right, and C5 on the left, and her sensory level of injury was C4 and C5, respectively, on the right and left sides. Her NLI was C4, her ASIA Impairment Scale score was C, and her SCIM III score was 20. Her ophthalmologic evaluation showed 3mm ptosis of the right upper eyelid (Figure 3) and miosis and anhidrosis on the same side. In dim illumination, the pupillary diameter on the right and left sides were 4mm and 5mm, respectively; in bright illumination, pupillary diameters were 3mm and 4mm on the right and left eye, respectively. Visual acuity, ocular alignment, ocular motility, fundus, iris, and cranial nerves were normal on both sides. The pupillary pharmacologic test suggested pre-ganglionic HS on the right side.

## Discussion

Johan Frederich Horner was the first to characterize the classic triad of HS, which consists of ptosis, miosis, and anhidrosis. HS results from damage to sympathetic fibres at any location along their path from the posterolateral hypothalamus to the CST [5]. The disruption of sympathetic innervation of the eye and ocular adnexa can occur at different levels. According to the disruption location, Horner syndrome's causes can be central, pre-ganglionic, or post-ganglionic [4].

The central (first-order) neurons are located in the posterolateral hypothalamus, and sympathetic fibres extend through the lateral brain stem to the Budge and Waller ciliospinal center in the intermediolateral grey column of the spinal cord at C8-T1. A central HS caused by damage to any of these structures is ipsilateral to the lesion, almost always unilateral, and frequently results in total body hemihypohidrosis. A central Horner syndrome may result from lesions in the lower cervical and upper thoracic spinal cord, including those caused by trauma, inflammatory or infectious myelitis, vascular malformations, demyelination, syrinx, syringomyelia, neoplasms, and infarction [6,7].

Pre-ganglionic sympathetic (second-order) neurons leave the Budge and Waller ciliospinal centre and cross the pulmonary apex. They then move up the carotid sheath and through the stellate ganglion to synapse at the superior cervical ganglion, situated at the level of the common carotid artery's bifurcation and the jaw's angle. Pre-ganglionic HS after trauma may be caused by apical lung lesions, damage to the brachial plexus or spinal roots, pneumothorax, first rib fracture, or neck hematoma, among other potential causes. Additionally, iatrogenic injury can harm the pre-ganglionic sympathetic chains [6]. The superior cervical ganglion is the site of origin of the post-ganglionic (third-order) sympathetic neurons, which travel through the internal carotid artery wall and onto the cavernous sinus. The fibres briefly travel with the abducens nerve within the cavernous sinus before joining the ophthalmic division of the trigeminal nerve and entering the orbit via its nasociliary branch. To travel with the lateral and medial suprachoroidal vascular bundles, reach the anterior segment of the eye, and innervate the iris dilator muscle, the sympathetic fibres in the nasociliary nerve split into two long ciliary nerves. Post-ganglionic HS is caused by lesions of the internal carotid arteries and an intraoral penetrating lesion [6,8,9].

A lesion to the CST can happen during surgery with an anterior or anterolateral approach to the cervical spine, causing pre-ganglionic damage to the sympathetic pathway to the eye. Additionally, extensive cervical dissection used to reveal the transverse foramen, uncovertebral joint, or lateral border of the cervical body can damage CST [10]. The CST passes over the longus colli muscle as it moves posteromedially toward the carotid sheath, convergent medially at C6, where the longus colli diverges laterally. This muscle is the longest and most medial of the prevertebral muscles, running from the anterior tubercle of the atlas to the bodies of the C3 to T3 vertebrae. The CST has located an average of 11.6 mm lateral to the medial border of the longus colli muscle at the level of C6 and closer to the medial border of the longus colli muscle at the C6 level than the C3 level. This proximity makes the CST more vulnerable to injury with a surgical approach to the lower cervical levels [11,12]. As in our scenario, the patient underwent a right anterior approach for ACDF at the C5 and C6 vertebrae level.

An injury to the CST may be due to forceful retraction of the longus colli muscle at C5-C6, transversely cutting of the longus colli muscle, or dissection of the prevertebral fascia [4]. It can be avoided by limiting the use of electrocautery along the lateral border of the vertebral body and through the longus colli, sub-periosteal dissection, and fixing the retractors beneath the edge of the longus colli muscle, also by avoiding forceful and prolonged retraction, during surgery [13,14].

The resolution rate of HS after ACDF ranges between 80%-100%. The average time to the appearance of improvement was 4.3 weeks, and the average time for complete resolution was six weeks. The outcome of HS resolution depends on the type of damage; if the damage was caused by retraction, there is often a spontaneous improvement and complete recovery. However, if there is complete sectioning of the sympathetic fibres, HS will not improve. The resolution of HS depends on the type of damage to the sympathetic trunk during surgery. If it was due to retraction, improvement and complete recovery are possible. But, complete sectioning of CST results in the persistence of symptoms [13,2]. In our case, HS persists even after one year of surgery. But then, symptoms like ptosis are not limiting the vision, and the patient did not find the ptosis to be a cosmetic burden.

## Conclusions

This case report focuses on the incidence of HS as an anterior cervical spine surgery complication. However, HS resulting from surgical injury to the ipsilateral CST is a rare complication of anterior spine surgery due to the anatomical proximity of the longus colli muscle and the cervical sympathetic trunk at the lower cervical level. Therefore, thoroughly understanding this anatomy, maintaining a midline position during the anterior approach, and avoiding forceful and prolonged retraction of the longus colli muscle may reduce the risk of CST and HS injury. In addition, the symptoms are temporary clinical manifestations in most cases, which are managed conservatively but may sometimes be irreversible complications.
